# A Prospective Investigation of the Impact of Distinct Posttraumatic (PTSD) Symptom Clusters on Suicidal Ideation

**DOI:** 10.1007/s10608-016-9829-2

**Published:** 2017-01-17

**Authors:** Maria Panagioti, Ioannis Angelakis, Nicholas Tarrier, Patricia Gooding

**Affiliations:** 1 Division of Population Health, Health Services Research & Primary Care, University of Manchester, Williamson Building, Oxford Road, Manchester, M13 9PL UK; 2 School of Psychology, University of South Wales, Pontypridd, UK; 3 Institute of Psychiatry, Kings College London, London, UK; 4 School of Psychological Sciences, University of Manchester, Manchester, UK

**Keywords:** Suicidal ideation, PTSD symptom clusters, Defeat/entrapment, Severity of depressive symptoms

## Abstract

Inconsistent findings have been reported by previous cross-sectional studies regarding the association between specific posttraumatic stress disorder (PTSD) symptom clusters and suicidality. To advance the understanding of the role of specific PTSD symptoms in the development of suicidality, the primary aim of this study was to investigate the predictive effects of the three specific PTSD symptom clusters on suicidal ideation prospectively. Fifty-six individuals diagnosed with PTSD completed a two-stage research design, at baseline and 13–15 months follow-up. The clinician administered PTSD scale (CAPS) was used to assess the severity of the PTSD symptom clusters and validated self-report measures were used to assess suicidal ideation, severity of depressive symptoms and perceptions of defeat entrapment. The results showed that only the hyperarousal symptom cluster significantly predicted suicidal ideation at follow-up after controlling for baseline suicidal ideation, severity of depressive symptoms and perceptions of defeat and entrapment. These findings suggest that both disorder-specific and transdiagnostic factors are implicated in the development of suicidal ideation in PTSD. Important clinical implications are discussed in terms of predicting and treating suicidality in those with PTSD.

## Introduction

The link between a diagnosis of posttraumatic stress disorder (PTSD) and suicidal ideation is well-established in the literature (Jakupcak et al. 2010; Panagioti et al. 2009, 2012; Richardson et al. 2012; Tarrier and Gregg 2004). Suicidal ideation in PTSD is associated with increased rates of subjective distress and disproportionally heightened rates of healthcare utilization (Chan et al. 2009; Bell and Nye 2007; Schnurr et al. 2000). Suicidal ideation is also a strong predictor of subsequent suicide attempts (Miranda et al. 2014; Simon et al. 2016). Thus, the identification of the best predictors of suicidal ideation in PTSD, which could be targeted by psychological suicide-prevention interventions, is of critical importance.

Based on previous developments, it has been emphasized that suicidal ideation in PTSD is potentially driven by both trans-diagnostic and PTSD specific features (Bolton et al. 2007; Johnson et al. 2008). To this end, important progress has been made towards understanding the role of generic (trans-diagnostic) factors that drive suicidal ideation in PTSD (Blake et al. 1995; Panagioti et al. 2012). In contrast, the role of PTSD-specific factors, such as distinct PTSD symptom clusters, in the development of suicidal ideation is less clear. Demonstrating that specific PTSD symptoms are strongly associated with suicidal ideation compared to others has the potential to facilitate the prompt identification of those individuals with PTSD who are at the highest risk for attempting suicide. So far, the studies that investigated the effects of specific PTSD symptoms on suicidal ideation have yielded inconsistent outcomes. Among veterans, two studies demonstrated that only the re-experiencing symptom cluster was significantly associated with suicidal ideation (Bell and Nye 2007) and acquired capacity for suicide (Bryan and Anestis 2011), whereas another study (Guerra et al. 2011) showed that suicidal ideation was uniquely positively associated with heightened levels of numbing symptoms. In addition, evidence was obtained that depression accounts for the impact of PTSD symptom clusters on suicidal ideation in veterans (Hellmuth et al. 2012). Among community research samples, Ben-Ya’acov and Amir (2004) showed that suicidal risk was predicted by high levels of hyperarousal symptoms and low levels of avoidance/numbing symptoms, whereas another study showed that hyperarousal and avoidance/numbing symptom clusters were associated with suicidal ideation in child Earthquake survivors even after controlling for depression symptoms (Chan et al. 2016). However, there are two key methodological limitations within the extant literature regarding the association between particular PTSD symptoms and suicidal ideation and behaviors, which could explain the above discrepancies. First, these studies were based on different populations and the direct comparison of their results is difficult (some were based on veterans, others on clinical populations, or community-based populations). Second, all the previous studies employed cross-sectional designs to investigate the link between specific PTSD symptom clusters and suicidal ideation. The above limitations suggest that there is a need for further studies using a prospective methodology to investigate the effects of specific PTSD symptom clusters on suicidal ideation.

Furthermore, a related unresolved issue concerns whether or not PTSD-specific factors contribute to suicidal ideation over and above the effects of generic and trans-diagnostic psychological factors. A newly developed psychological perspective of suicide, the Schematic Appraisals Model of Suicide (SAMS; Johnson et al. 2008), provides a useful theoretical framework, which could explain the psychological mechanisms of suicidal ideation in PTSD. The SAMS model is a re-conceptualization of a highly influential model of suicide, the Cry of Pain Model of Suicide (Williams 1997; Williams et al. 2005). The advance of the SAMS model over its predecessor is that it has highlighted the importance of a personal cognitive appraisal system (e.g. an internal network that incorporates the individual’s subjective beliefs, judgments and attitudes towards the self, the world and the others) in the development of suicidal thoughts and behaviors (Panagioti et al. 2012). A category of negative appraisals, central to the SAMS, refers to defeat and entrapment, which are viewed as the proximal mechanism to suicidal ideation (Johnson et al. 2008). Consistent with this view, a number of recent cross-sectional studies has demonstrated that defeat/entrapment is strongly associated with suicidal ideation in a range of research populations including those with PTSD (Panagioti et al. 2012; Taylor et al. 2010, 2011). In addition to defeat/entrapment, another well-established generic predictor of suicidal ideation in PTSD is comorbid depressive symptoms (Bryan and Corso 2011; Clover et al. 2004; Menard 1995; Panagioti et al. 2009).

The aim of this study was to examine whether the three PTSD symptom clusters predict suicidal ideation prospectively (13–15 months later). To ensure that the effects of the specific PTSD symptom clusters on suicidal ideation are not an artifact of the effects of other key generic predictors of suicidal ideation, we accounted for baseline suicidal ideation, perceptions of defeat and entrapment and severity of depressive symptoms in the analyses. The SAMS argues that perceptions of defeat and entrapment should be best represented as a single construct, because they are conceptually synonymous and originate from the same cognitive mechanism (a negatively biased appraisal system; Johnson et al. 2008; Taylor et al. 2011). This suggestion has also been confirmed by a study that employed an explanatory factor analysis in a student population (Taylor et al. 2009). Therefore, consistent with our previous work (Panagioti et al. 2012; Schnurr et al. 2000; Taylor et al. 2010a, b), defeat and entrapment in this study was conceptualized as a single construct (referred to as defeat/entrapment hereafter).

## Method

### Participants

Participants were recruited using adverts (i.e., newspaper advertising, online advertising, posters in NHS mental health services based in Manchester, and voluntary agencies) asking for people who had experienced a traumatic event in the past, and have been affected by it, to volunteer. Potential participants were sent by post or email a self-report measure, the posttraumatic stress diagnostic scale (PDS; Foa et al. 1997) to assess whether they met the inclusion criteria for the study. Those participants who returned the PDS scale and met the inclusion criteria of the study proceeded to the full assessment. Participants had to fulfill the following criteria to participate in the first stage of the study: (1) they had to have experienced a serious traumatic event in the past and meet criterion A of the PDS scale (Foa et al. 1997). This inclusion criterion was used to ensure that all the prospective participants had been exposed to a traumatic experience that was severe enough to meet the criterion A of the PDS scale, (2) be aged between 18 and 65 years, (3) fulfill the criteria for a lifetime diagnosis of PTSD confirmed by the Clinical Administrated PTSD scale (CAPS) for DSM IV (Blake et al. 1995), (4) have experienced at least one re-experiencing symptom in the past month with ≥1 frequency and ≥2 intensity scores determined by the CAPS, and (5) have a thorough grasp of the English language (this was necessary for participation in the assessment interview and for the understanding of questionnaire items). In addition, participants had to provide informed consent and be willing to come at the University of Manchester to carry out the study. Participants were excluded if they suffered from dementia, organic brain disorder or an active psychotic disorder. Two individuals were excluded in the initial phase because they reported suffering from psychosis. In order to participate in the second stage of the research (follow-up), potential participants had to successfully complete the first stage of the study and to have provided their consent (at the baseline session) to be re-contacted for the second stage of the research. All participants received treatment by NHS primary care (e.g. their General Practitioner and/or Primary Care Mental health teams) or secondary mental health services. With participant consent, service providers were notified about their patients’ participation in the study.

### Measures

#### PTSD

The CAPS for DSM-IV (Blake et al. 1995) was used to confirm a diagnosis of PTSD (current or lifetime) and to assess the current severity of the three individual symptom clusters. It consists of 30 items, which are scored in 5-point Likert scale, ranging from 0 = *never* to 4 = *nightly or almost every night*. In addition to producing a total PTSD severity score, CAPS also produces severity scores for the three PTSD symptom clusters, namely the re-experiencing symptom cluster, the avoidance/numbing symptom cluster and the hyperarousal symptom cluster. The severity scores for each symptom cluster were computed by adding the intensity and frequency scores for each of the PTSD symptom cluster. Previous research has found that the alpha coefficient ranges from 0.85 to 0.87 for the three symptom clusters (Blake et al. 1995; Weathers et al. 2001). In this study, the alpha coefficient for re-experiencing, avoidance/number and hyperarousal symptom clusters were 0.88, 0.87 and 0.87, respectively.

#### Depression

The Beck Depression Inventory (BDI-II; Beck et al. 1996) comprises 21 items, which measure the severity of depressive symptoms using a 4-point scale (range 0–63) in the past 2 weeks. The BDI has high internal consistency (*α* = 0.86 for psychiatric patients and *α* = 0.81 for non-psychiatric individuals) and concurrent validity with respect to clinical ratings and high agreement with the Hamilton Psychiatric Rating Scale for Depression (HRSD) for psychiatric (0.72 and 0.73, respectively) and non-psychiatric individuals (0.60 and 0.74 respectively; Beck et al. 1996; Dozois et al. 1998; Richter et al. 1998). In this sample, the alpha coefficient was 0.94. The item 9 of the BDI (referring to suicidal ideation and intent) was removed from the total BDI score used in the multiple regression analyses examining predictors of suicidal ideation.

#### Defeat and Entrapment

The defeat scale (Gilbert and Allan 1998) consists of 16 items assessing perceptions of defeat, including those of failed struggle and low social rank in the past week. The items are rated on a 5-point scale, ranging from 0 = *never* to 4 = *always*/*all the time*. Higher scores indicate greater feelings of defeat. The alpha coefficient for this scale has been found to be 0.94 in a student group and 0.93 in a depressed group (Gilbert and Allan 1998). In this sample, the alpha coefficient of the scale was 0.94. The entrapment Scale (Gilbert and Allan 1998) consists of 16 items assessing perceptions of being trapped by external or internal stressors. The items are rated on a five-point scale ranging from ‘Not at all like me’ to ‘Extremely like me’. The alpha coefficient for this scale has been found to range from 0.86 to 0.89 in a depressed group (Gilbert and Allan 1998) and it was 0.95 in the present sample.

#### Suicidal Ideation

The Suicidal Behaviors Questionnaire-Revised (Osman et al. 2001) is a 4-item measure, which assesses different levels of suicide risk, including lifetime suicidal thoughts and behaviors, suicidal ideation in the past year, communication of suicide intent and the likelihood of committing suicide in the future. The total score ranges from 3 to 18 with higher scores indicating greater levels of suicide risk. In this study only the second item of the SBQR was utilized to assess suicidal ideation (“how often have you thought about killing yourself in the past year”). Responses on this item range from 1 = never to 5 = very often. The test–retest reliability of the second item of the SBQ—R was 0.83 in the present sample.

### Procedure

Participants were administered the clinician administered PTSD scale (CAPS) and a series of standardized self-report questionnaires to assess defeat/entrapment, severity of depressive symptoms and suicidal ideation at baseline. Thirteen to fifteen months later, all the participants (after giving their consent to be re-contacted) were re-invited to participate in the second stage of the research (follow-up). At follow-up, participants again completed the suicidal ideation measure. Both at baseline and follow-up, the CAPS and the self-report measures were administrated by the first author (MP) in one session. Ethical approval for this research has been obtained by the relevant NHS Research Ethics Committee in North West of England.

### Data Analyses

Initially, the variables to be included in the analyses were screened for skew and multicollinearity. Defeat, entrapment, depression symptom severity and suicidal ideation were significantly positively skewed. There was no multicollinearity problem since all the tolerance values were above 0.2 (Menard 1995). Zero order correlations were carried out to examine the relationships among all the variables of the study. Subsequently, multiple hierarchical regression analyses were conducted to examine which PTSD symptom cluster(s) at baseline would predict suicidal ideation at follow-up. Defeat and entrapment were combined and entered in the regression analyses as a single variable in accordance with findings that suggest that defeat and entrapment are better conceptualized as a single factor rather than two separate ones (Taylor et al. 2009). We performed 1000 bootstrap replications of the original data to account for skew and test the robustness of the analyses. All analyses were conducted using SPSS statistical version 22.

## Results

### Descriptive Characteristics

Overall, 73 participants were recruited for participation but 17 of those failed to participate at follow-up. Thus, the analyses were based on the remaining 56 individuals (*M*_*age*_ = 28.32, *SD* = 10.83) who reported current or lifetime PTSD, as shown in Table 1. The majority of the participants were female (*n* = 44, 78.6%), unmarried (*n* = 37, 66.1%) and white (*n* = 42, 75%). 39 (69.6%) individuals fulfilled the criteria for a current PTSD diagnosis, whereas 17 individuals fulfilled the criteria for a lifetime PTSD diagnosis. The vast majority of the participants who completed the follow-up study, had met the CAPS criteria for a current PTSD diagnosis during the baseline assessment which was undertaken 13–15 months earlier (*n* = 46 of the 56 participants, 82%). As many as 37 (66.1%) participants had experienced lifetime suicidal behaviors, whereas 30 (53.6%) participants reported suicidal ideation in the past month. Physical and sexual abuse was reported as the most frequent trauma experienced by the participants (*n* = 28), followed by accident/hazard (*n* = 19). War trauma (*n* = 6) and ‘other’ traumas (*n* = 3) were reported only by the minority of the study sample. The means, standard deviations and zero order correlations among the core variables of the study are presented in Table 2.

**Table 1:** Descriptive and clinical characteristics of the study sample

	*N*	%
Gender		
Male	12	21.4
Female	44	78.6
Marital status		
Unmarried	37	66.1
Co-habituating	19	33.9
Ethnicity		
White	42	75
Black/Asian	14	25
Type of PTSD		
Current	39	69.6
Lifetime	17	30.4
Type of suicidality		
Behaviors	37	66.1
Ideation	30	53.6
Type of trauma		
Sexual abuse	28	50
Accident/hazard	19	33.9
War trauma	6	10.7
Other traumas	3	5.4

	*M*	*SD*
Age	28.32	10.83
PTSD severity (CAPS)	55.97	21.36
Depression (BDI-II)	21.61	11.08
Defeat scale	20.13	14.16
Entrapment scale	17.19	13.86

*PTSD* post-traumatic stress disorder, *CAPS* clinician administered PTSD scale, *BDI-II* Beck Depression Inventory II

**Table 2:** Means, standard deviation and correlations for the variables in the model

	Mean (*SD*)	2	3	4	5	6	7	8
1. Re-experiencing symptom cluster (CAPS) at baseline	14.65 (7.49)	0.80**	0.74**	0.68**	0.64**	0.75**	0.64**	0.60**
2. Avoidance/numbing symptom cluster (CAPS) at baseline	17.87 (11.83)		0.82**	0.78**	0.79**	0.86**	0.76**	0.67**
3. Hyperarousal symptom cluster (CAPS) at baseline	14.48 (8.50)			0.67**	0.66*	0.73**	0.59**	0.67**
4. Defeat at baseline	20.13 (14.16)				0.88**	0.87**	0.72**	0.74**
5. Entrapment at baseline	17.19 (16.54)					0.87**	0.79**	0.79**
6. Severity of depressive symptoms (BDI-II) at baseline	17.13 (13.86)						0.81**	0.83**
7. Suicidal ideation (SBQ-R item 2) at baseline	2.04 (1.41)							0.84**
8. Suicidal ideation (SBQ-R item 2) at follow-up	2.17 (1.42)							

**p < 0.01

### Regression Analyses

A multiple regression was conducted to examine whether the three PTSD symptom clusters at baseline would predict suicidal ideation at follow-up after controlling for suicidal ideation, defeat/entrapment and the severity of depressive symptoms at baseline. Suicidal ideation, defeat/entrapment and severity of depressive symptoms at baseline were entered in the first step of the regression analyses and the three PTSD symptom clusters at baseline were entered in the second step of the regression analyses. Suicidal ideation at follow-up was the outcome measure in the regression analyses. Following the addition of the three PTSD symptom clusters, the overall regression model was significant *F* (6, 48) = 45.44, *p* < 0.001. However, only the hyperarousal symptom cluster significantly predicted suicidal ideation at follow-up whilst controlling for baseline suicidal ideation and severity of depressive symptoms. The statistics of the regression analyses are presented in Table 3.

**Table 3:** Results from the regression analyses examining the three PTSD symptom clusters at baseline as predictors of suicidal ideation at follow-up

Step	Variable entered	B	SE	Total R^2^(*f* ^2^)	ΔR^2^(*f* ^2^)
1	Baseline suicidal ideation	0.504***	0.023	0.75*** (0.30)	
	Severity of depressive symptoms	0.021**	0.010		
	Defeat/entrapment	0.044**	0.012		
2	Baseline suicidal ideation	0.422***	0.109	0.82** (0.46)	0.07** (0.08)
	Severity of depressive symptoms	0.022*	0.011		
	Defeat/entrapment	0.028**	0.011		
	Re-experiencing symptom cluster	0.008	0.020		

*p < 0.05; **p < 0.01; ***p < 0.001

## Discussion

The aim of this study was to examine the effects of distinct PTSD symptom clusters on suicidal ideation using a prospective design. The outcomes of the analyses showed that the hyperarousal symptom cluster was the only PTSD symptom cluster that predicted suicidal ideation 13–15 months later. This study advances the extant literature regarding the link between the distinct PTSD symptom clusters and suicidal ideation in two ways. The first key advance of this study is that it tested prospectively the effects of the three specific PTSD symptom clusters on suicidal ideation. Hyperarousal was the only PTSD symptom cluster that predicted suicidal ideation 13 to 15 months later. Ben’Yacov and Amir (2004) also showed that only the hyperarousal, but not the other two symptom clusters, predicted suicide risk in a community male sample. This finding suggests that hyperarousal symptom cluster is the most critical PTSD symptom cluster in relation to suicidal ideation and emphasizes the need for continuously monitoring and therapeutically targeting hyperarousal symptoms to prevent suicidal ideation in people with PTSD symptoms.

The second key advance of this study was that it demonstrated that the hyperarousal symptom cluster prospectively predicted suicidal ideation even after accounting for the effects of three key trans-diagnostic predictors of suicidal ideation in PTSD including perceptions of defeat/entrapment, severity of comorbid depressive symptoms and past suicidal ideation. Although the findings of this study are in agreement with the existing theoretical (SAMS and Cry of Pain model) and research evidence, which has shown that perceptions of defeat/entrapment and comorbid depression are core psychological processes implicated in suicidal ideation (as indicated by the significant regression effects; Johnson et al. 2008; Panagioti et al. 2014; Williams et al. 2005), it further suggests that the contribution of the severity of hyperarousal symptom cluster on suicidal ideation is equally important to that of the above generic factors and therefore there are also PTSD-specific factors that are implicated in the process of suicide thinking. It should be noted, however, that hyperarousal symptoms such as physiological arousal, anxiety, insomnia and poor concentration are considered core elements of the PTSD experience, but they could also be seen as trans-diagnostic features because they are also present in other mood and anxiety disorders. Theories such as the diathesis-stress model and the positive feedback model for suicide emphasize the role of hyperarousal in the development of suicidal ideation and have received empirical support in other research populations including people with substance use disorders and depression (Capron et al. 2016; Katz et al. 2011). These models could be useful frameworks for understanding and preventing suicidal ideation in PTSD. Another possibility is that other unmeasured factors such as perceptions of guilt, self-deprecation and self-consciousness, which are identity-based cognitions, best account for the experience of suicidal ideation in people with PTSD symptoms compared to perceptions of defeat/entrapment, which are mainly situational cognitions. Consistent with this hypothesis, a number of cross-sectional studies found that feelings of guilt, shame and self-deprecation mediated the effects of PTSD symptoms on suicidal ideation (Bryan et al. 2013; Carr et al. 2013). Prospective studies are needed to examine these potentially important mechanisms of suicidal ideation in PTSD. In particular, the investigation of the unique effects of several theory-driven trans-diagnostic factors and PTSD-specific factors has the potential to significantly advance the understanding of the main drivers of suicidal ideation in PTSD. This approach will allow us to draw more definitive conclusions of whether suicidal ideation in PTSD is primarily driven by trans-diagnostic, disorder-specific or comorbid factors or by an amalgam of these three factors (Bolton et al. 2007; Guerra et al. 2011; Jurisic and Marusic 2009; O’Connor et al. 2013; Schnurr et al. 2000; Williams 1997). With regards to clinical practice, these findings suggest that to facilitate the accurate identification and therapeutic management of suicide risk in PTSD, a multi-target strategy, which would focus simultaneously on the severity of perceptions of defeat/entrapment, the severity of the hyperarousal symptoms, and the severity depressive symptoms, is likely to be the most productive.

A number of limitations of this study warrant discussion. First, this study only accounted for the effects of baseline suicidal ideation and depressive symptoms in the analyses. Whether these findings persist over and above the effects of other important risk factors for suicidal ideation (i.e., hopelessness, no rescue, perfectionism, perceived burdensomeness and thwarted belongingness; Joiner and Van Orden 2008; O’Connor et al. 2010; Williams et al. 2005) need to be addressed by subsequent studies. Second, the sample of the study was relatively small. However, the analyses clearly demonstrated that the hyperarousal symptom cluster significantly predicted suicidal ideation 13 or 15 months later. Third, this study only examined the effects of distinct PTSD symptom clusters on suicidal ideation, but not on other suicidal behaviors, such as suicide attempts. More research is needed to examine whether the same pattern of findings is evident among PTSD individuals with a history of suicide attempts. Although suicidal ideation and suicide attempts often co-exist, contemporary theoretical and empirical evidence suggests that different psychological mechanisms might be implicated in the development of suicidal ideation and suicide attempts (Wyder and De Leo 2007). According to ‘the ideation to action’ framework, pain and hopelessness are the main factors that lead suicidal ideation, whereas acquired capacity for suicide is the strongest predictor of suicide attempts beyond the effects of suicidal ideation (Klonsky and May 2015). Fourth, a single 5-point scale, the second item of the SBQR-II measure, which has sound psychometric properties (Taylor et al. 2010), was used to measure suicidal ideation in this study. We chose this measure because it is brief, easy to complete, and fitted well with the time-line of the study. Although previous investigations have also utilized one-item measures to assess suicidal ideation (Casey et al. 2008; Fialko et al. 2006), the future use of more extensive measures of suicidal ideation, would be beneficial. Fifth, in this study, we included participants with a current diagnosis of PTSD and also participants who were diagnosed with PTSD in the past and were experiencing subthreshold PTSD symptoms at present. This decision was based on evidence that the experience of subthreshold symptoms of PTSD is associated with significant functional impairment and comparable rates of suicidal behaviors with a full current diagnosis of PTSD (McLaughlin et al. 2015; Zlotnick et al. 2002). It has also been suggested that restricting the research and clinical focus to people with a full PTSD diagnosis is likely to hamper treatment access for people with subthreshold but clinically significant PTSD symptoms (Greenberg et al. 2015; Grubaugh et al. 2005). As a sensitivity analysis, we repeated the analyses in the subsample of the 39 people who had a current diagnosis of PTSD at baseline and completed the follow-up assessment measures. We noted no changes in the results. However, future larger studies on people with a current diagnosis of PTSD would be useful to replicate these findings. Finally, the DSM-IV criteria were used in assigning a PTSD diagnosis and in classifying the PTSD symptoms into three distinct clusters. In DSM-5, a number of major revisions were introduced. The most relevant revision for the results of this study is the division of the PTSD symptoms into 4 clusters in DSM 5 as opposed to 3 clusters in DSM-IV and the addition of three new symptoms. The re-experiencing cluster remained the same and only one change was introduced to the hyperarousal cluster (one symptom assessing the presence of “reckless or destructive behavior” was added), which was found to be the most important predictor of suicidal ideation in this study. Given the minor changes on the arousal cluster and the commonalities of the newly introduced symptom of recklessness and destructive behavior with the concepts of impulsivity and self-harm (which are highly correlated with suicidal ideation), we assume that the effects of the arousal and the re-experiencing clusters on suicidal ideation would be the same if the DSM-5 criteria had been used. However, the avoidance/numbing symptom cluster was divided into two separate clusters, the avoidance cluster and negative alterations in cognition and mood cluster, and two new symptoms were introduced to the latter cluster. These extensive changes do not allow any assumptions about the effects of these two revised symptom clusters on suicidal ideation. We certainly recommend the replication of these findings by future studies using the DSM-5 framework.

Despite the limitations above, this is the first study that demonstrated, using a prospective design, that the hyperarousal is the only PTSD symptom cluster which prospectively predicts suicidal ideation. Overall, suicidal ideation in PTSD appears to be a multifaceted phenomenon which is likely to be driven by an amalgam of trans-diagnostic factors, including defeat/entrapment and comorbid factors, such as severity of depressive symptoms and the hyperarousal symptom cluster. Future larger studies are encouraged to confirm and expand these findings.
